# AI-based retrospective analysis: differential improvement profiles of medication and deep brain stimulation in Parkinson's disease

**DOI:** 10.3389/fneur.2026.1758218

**Published:** 2026-03-13

**Authors:** Lu Su, Aiwen Li, Zhanxu Li, Yilin Liu, Geng Cheng, Bo Shen, Jian Wang, Jianjun Wu

**Affiliations:** 1 Department of Neurology and National Research Center for Aging and Medicine & National Center for Neurological Disorders, State Key Laboratory of Brain Function and Disorders, Huashan Hospital, Fudan University, Shanghai, China; 2 NERVTEX Co., Ltd, Shanghai, China; 3 NERVTEX Co., Ltd, Wuhan, China

**Keywords:** artificial intelligence, deep brain stimulation, levodopa, multidimensional assessment, Parkinson's disease

## Abstract

**Background:**

Bradykinesia in Parkinson's disease (PD) involves reduced movement speed, amplitude, and rhythmicity. While the MDS-UPDRS Part III is the standard clinical tool for motor assessment, it has limited sensitivity to specific kinematic features. Levodopa and subthalamic nucleus deep brain stimulation (STN-DBS) are common treatments for PD, yet their differential effects across motor domains are not fully characterized. This study applies AI-based video analysis to evaluate the effects of levodopa and STN-DBS on limb bradykinesia.

**Methods:**

This retrospective study assessed fifty-three patients with Parkinson's disease undergoing STN-DBS. Motor performance was video-recorded during Levodopa-off and Levodopa-on states (levodopa challenge test performed prior to surgery), as well as after DBS activation (OFF_MED_/OFF_STIM_, OFF_MED_/ON_STIM_, ON_MED_/ON_STIM_). Both clinical assessments and subsequent video-based analyses focused on the MDS-Unified Parkinson's Disease Rating Scale (MDS-UPDRS), Part III, specifically evaluating items 3.4 Finger Tapping, 3.5 Fist-clenching test, 3.7 Toe Tapping, and 3.8 Leg Agility. Motor function was first evaluated using conventional UPDRS-III item scores rated by two experienced specialists, with the primary clinical comparison defined between the levodopa-on and OFF_MED_/ON_STIM_ states, to explore the differential therapeutic emphases of medication and DBS. Subsequently, AI-based video analysis was applied to quantify kinematic parameters, including amplitude, frequency, and coefficients of variation, using AI algorithms (NERVTEX Co. Ltd.). Comparisons were made for levodopa effects (Levodopa-off vs. Levodopa-on), DBS effects (OFF_MED_/OFF_STIM_ vs. OFF_MED_/ON_STIM_), and therapy-specific differences (Levodopa-on vs. OFF_MED_/ON_STIM_).

**Results:**

Conventional UPDRS-III item scores suggested that levodopa was more effective than DBS in improving upper-limb tasks (items 3.4 Finger Tapping and 3.5 Fist-clenching test), while lower-limb tasks (items 3.7 Toe Tapping and 3.8 Leg Agility) showed no significant changes. In contrast, AI-based kinematic analysis revealed more differentiated treatment effects. Levodopa was associated with improvements in movement speed, amplitude, and stability in the upper limbs, as well as a significant impact on lower-limb amplitude, both in toe tapping (item 3.7) and leg agility (item 3.8). DBS, by comparison, enhanced upper-limb motor output but had limited effects on the lower limbs, with improvements in speed and amplitude observed only in the toe tapping (item 3.7) task. Additionally, levodopa demonstrated superior improvements in lower-limb amplitude, both in toe tapping (item 3.7) and leg agility (item 3.8), compared to DBS.

**Conclusion:**

This study demonstrates that AI-based kinematic analysis enables a nuanced and individualized characterization of motor responses to medication and STN-DBS in Parkinson's disease, complementing conventional clinical scoring. Although both therapies improve bradykinesia, they appear to preferentially modulate distinct motor domains across individuals, underscoring their complementary roles in treatment. These findings highlight the potential of AI-based motor assessment to support personalized symptom profiling and more individualized therapeutic decision-making in Parkinson's disease.

## Background

Parkinson's disease (PD) is clinically defined by bradykinesia, and/or resting tremor, rigidity, with bradykinesia being a multidimensional symptom encompassing slowness, reduced movement amplitude, and dysrhythmia (1). The Unified Parkinson's Disease Rating Scale (UPDRS), particularly Part III (UPDRS-III) (2, 3), is widely used to assess motor dysfunction in clinical practice. However, as a clinician-rated, ordinal scale, UPDRS-III may have limited sensitivity to subtle differences in motor patterns, including changes in movement amplitude, frequency, and other fine-grained kinematic features.

Levodopa remains the gold standard for pharmacological treatment, providing effective relief from motor symptoms, especially in the early stages of the disease. With disease progression, patients commonly develop motor fluctuations (4). To sustain symptom control, increasing levodopa dosing is often required, which may lead to levodopa-induced dyskinesia (LID), and other treatment-related complications. In contrast, deep brain stimulation (DBS) targeting the subthalamic nucleus (STN) has emerged as an effective surgical option for patients with inadequate or complicated responses to medication. STN-DBS has been demonstrated to reduce motor symptoms and decrease the need for medications in some patients.

Although both levodopa and STN-DBS are effective in alleviating cardinal motor symptoms such as tremor, rigidity, and bradykinesia (5, 6), accumulating evidence suggests that their therapeutic effects are not uniform across motor domains. In particular, differences have been reported in their modulation of movement amplitude, rhythm, and task-specific motor control (7). These findings underscore the need for approaches capable of differentiating treatment-specific motor response profiles beyond conventional clinical scoring. Recent advances in artificial intelligence—particularly computer vision (CV)—have made it feasible to extract quantitative kinematic information from standard RGB videos (8–10). Accordingly, CV-based movement assessment is increasingly being translated from laboratory research into clinical workflows for neurological conditions. In brief, pose estimation is applied frame-by-frame to obtain anatomical keypoint coordinates, from which objective kinematic features are derived; in some implementations, these features are further combined with machine learning models to estimate clinical ratings or detect pathological movement patterns. The clinical impetus for this approach lies in its ability to reduce subjectivity inherent to manual scoring, lower the cost and operational barriers to quantitative assessment, and improve ecological validity by enabling longitudinal, often remote, monitoring of functional status and treatment response in naturalistic settings.

Against this background, this study aims to leverage AI-driven video analysis to objectively evaluate the effects of levodopa and STN-DBS on specific motor tasks in PD patients, focusing on UPDRS-III items such as finger tapping (item 3.4), fist-clenching test (item 3.5), toe tapping (item 3.7), and leg agility (item 3.8). By utilizing AI analysis, this research intends to provide more accurate and reliable quantification of motor characteristics, revealing the distinct impacts of L-Dopa and STN-DBS on motor control in PD patients.

## Methods

Fifty-three PD patients who underwent STN-DBS participated in this study (Table 1). All participants provided informed consent in accordance with the Declaration of Helsinki prior to inclusion in the study. The study protocol was approved by the Institutional Review Board of Huashan Hospital, Fudan University (2011213).

**Table 1:** Demographic and clinical characteristics of the study participants.

**Characteristics**	***n* = 53**
Age, mean (SD)	62.15 (9.12)
Male, *n* (%)	26 (49.1%)
Disease duration (years), mean (SD)	9.87 (4.86)
Peak time (minutes), mean (SD)	60.40 (23.43)
H and Y, mean (SD)	3.14 (0.77)
Presurgical levodopa-equivalent daily dose (LEDD), mean (SD)	951.02 (293.21)
Total improvement rate (%), mean (SD)	50.15 (13.47)
Dyskinesia, *n* (%)	27 (50.9%)

Data are presented as mean (standard deviation, SD) for continuous variables and number (percentage, %) for categorical variables. H&Y, Hoehn and Yahr stage; LEDD, levodopa-equivalent daily dose. The table summarizes baseline demographic features, disease-related characteristics, medication exposure, and clinical outcomes of the study cohort.

All DBS surgeries were performed at the Department of Neurosurgery, Huashan Hospital, Fudan University, following standardized protocols to ensure consistency and accuracy (11). The surgical procedures followed standardized protocols consistent with established practice. Before surgery, all patients underwent high-resolution magnetic resonance imaging with a 3.0-T scanner (Discovery MR750w, GE Healthcare, Milwaukee, WI) to delineate the subthalamic nucleus (STN) and plan the stereotactic trajectory. Bilateral implantation of quadripolar electrodes (Model 3389, Medtronic, Minneapolis, MN; or PINS 301, Beijing PINS Medical Co., Beijing, China) was performed using MRI-guided stereotactic navigation combined with intraoperative microelectrode recordings (MER). MER was acquired through a five-channel recording system (Leadpoint 5, Medtronic) equipped with a Star-Drive microelectrode array, enabling physiological confirmation of STN boundaries and refinement of target localization. Following satisfactory electrophysiological mapping, electrodes were connected to an implantable pulse generator (Activa RC, Medtronic), which was placed subcutaneously. Test macrostimulation was applied along selected trajectories to verify optimal electrode positioning and guide final adjustment of stimulation parameters. Postoperative imaging (CT or MRI) was routinely performed to confirm electrode placement and to exclude procedure-related complications prior to DBS activation. No patients included in this study experienced major postoperative complications, such as symptomatic intracranial hemorrhage, that would have precluded DBS programming or motor assessment.

### Clinical assessment and video data collection

Clinical assessments were performed using the MDS-Unified Parkinson's Disease Rating Scale (MDS-UPDRS), Part III, focusing on items 3.4 finger tapping, 3.5 fist-clenching test, 3.7 toe tapping, and 3.8 leg agility. Two experienced specialists independently scored the videos, the final score were derived from their ratings. When discrepancies occurred between the two ratings, the average of the scores was used.

Each patient underwent a levodopa challenge test prior to surgery, during which video recordings were obtained in both the Levodopa-off and Levodopa-on states. Patients took their last dose of antiparkinsonian medication the night before, and motor assessments were performed the following morning in the medication-off state, corresponding to an approximate 12–14-h washout period. One month post-surgery, deep brain stimulation (DBS) was activated to allow for sufficient healing and stabilization (6). Neurologists conducted programming sessions to adjust the stimulation settings based on individual patient responses and clinical evaluations. During this phase, video recordings were made to document the patient's motor performance in three different conditions: no medication and stimulation (OFF_MED_/OFF_STIM_), STN-DBS only (OFF_MED_/ON_STIM_), STN-DBS and levodopa (ON_MED_/ON_STIM_).

Since this is a retrospective study utilizing videos retained during patient follow-up, some videos from patients are missing. All manual assessments were based on the available videos. To better assess the effects of medication and DBS, we determined the more affected side based on the Levodopa-off state scores and extracted the corresponding limb scores. When no significant side difference was observed, left-side scores were used for further analysis.

### AI-based multidimensional movement analysis methodology

Video-based motion analysis was performed using Movement Dysfunction Assessment Software (MoDAS v2.2.0, NERVTEX Co. Ltd., Wuhan, China). Specifically, deep learning–based pose estimation was applied to standard RGB videos to detect and track anatomical keypoints frame-by-frame, thereby converting raw video into task-specific kinematic time-series signals that reflect the movement of interest. Movement cycles were identified using peak–trough detection on the corresponding amplitude signal. For each detected cycle, we extracted cycle-wise amplitude (peak-to-trough excursion) and cycle-wise period. Kinematic features were summarized across cycles, including mean values and variability measures. Coefficient of variation (CoV) was computed across cycles as SD/mean for both amplitude and period. Lower CoV indicates greater stability. Rate of variation (RoV) was computed for both amplitude and period from the first 10 cycles as [mean (last third)—mean (first third)]/mean (first third); a negative RoV indicates worsening performance over repetitions, whereas a positive RoV indicates progressive improvement. The remaining tasks were analyzed using analogous procedures with task-specific signal definitions. Further details on the pose estimation and kinematic feature extraction methods have been reported previously (12).

### Research framework and analysis approach

As this was a retrospective study, the availability of video recordings varied across conditions. A total of 53 videos were available for both the Levodopa-off and Levodopa-on states obtained during the preoperative levodopa challenge test. For postoperative DBS assessments, 50 videos were available for the OFF_MED_/OFF_STIM_ condition, 52 for the OFF_MED_/ON_STIM_ condition, and 50 for the ON_MED_/ON_STIM_ condition. All available recordings were included in the AI-based analyses. Because certain subtle movements could not be reliably detected by the pose estimation algorithm, the effective sample size varied slightly across individual motor tasks.

Clinical motor performance was first evaluated using conventional UPDRS-III item scores rated by specialists, with the primary comparison defined between the Levodopa-on and OFF_MED_/ON_STIM_ states, to directly contrast the therapeutic effects of medication and DBS at the clinical scale level.

Subsequently, AI-based video analysis was applied to extract quantitative kinematic features from the same recordings. Using these features, comparisons were conducted to further characterize treatment-specific response patterns, including levodopa effects (Levodopa-off vs. Levodopa-on), isolated DBS effects (OFF_MED_/OFF_STIM_ vs. OFF_MED_/ON_STIM_), and therapy-specific differences (levodopa-on vs. OFF_MED_/ON_STIM_).

To better assess the effects of medication and DBS, we determined the more affected side based on the Levodopa-off state scores (scored by specialists) and extracted the corresponding limb scores and AI-based feature parameters. When no significant side difference was observed, left-side scores and kinematic features were used for further analysis.

### Data analysis and statistics

The data analysis was conducted using python. To compare the UPDRS III scores (specialists-based) across different patient states, the Wilcoxon rank-sum test was applied. For the analysis of all AI-derived kinematic features, normality tests were first performed. For features that followed a normal distribution, paired *t*-tests were used, while for features that did not meet the normality assumption, the Wilcoxon rank-sum test was applied.

Statistical analyses were primarily based on within-subject comparisons across treatment conditions, with each patient serving as their own control. No multivariable regression models or formal covariate adjustments were applied. *p*-values are reported as uncorrected, and no formal correction for multiple comparisons (e.g., Bonferroni or FDR) was performed, as the analyses were exploratory in nature.

## Results

### Conventional UPDRS scores show item-specific changes with limited sensitivity to treatment-specific effects

The upper-limb UPDRS-III items, including finger tapping and fist-clenching test, showed statistically significant but modest improvements in the OFF_MED_/ON_STIM_ state compared with the Levodopa-on state, whereas lower-limb items (toe tapping and leg agility) did not show significant changes (Table 2). Although these upper-limb differences reached statistical significance, the absolute mean differences were small and the associated *p*-values were borderline. Moreover, item-level score changes alone do not reveal which specific movement components, such as speed, amplitude, or rhythmicity, contributed to the observed improvement, as each UPDRS-III item integrates multiple kinematic aspects into a single ordinal score.

**Table 2:** Clinician-rated MDS-UPDRS Part III item scores (items 3.4, 3.5, 3.7, and 3.8; mean ± SD) comparing Levodopa-on and OFF_MED_/ON_STIM_ states.

**Variable**	Manual scoring (***n*** = 51)		
	**Levodopa-on state**	**OFF**_MED_ **/ON**_STIM_	***p*** **-value**
3.4 finger tapping	1.33 ± 0.67	1.61 ± 0.84	0.04^*^
3.5 fist-clenching test	1.20 ± 0.52	1.45 ± 0.81	0.04^*^
3.7 toe tapping	1.63 ± 0.64	1.84 ± 0.73	0.11^ns^
3.8 leg agility	1.22 ± 0.41	1.29 ± 0.61	0.50^ns^

Values are presented as mean ± standard deviation (SD). Manual scoring was performed for MDS-UPDRS Part III items 3.4 (finger tapping), 3.5 (hand movements/fist-clenching), 3.7 (toe tapping), and 3.8 (leg agility). P-values represent comparisons between Levodopa-on and OFF_MED_/ON_STIM_ states. ^*^p < 0.05; ^**^p < 0.01; ^***^p < 0.001; ns, not significant. These results reflect differential motor performance across pharmacological and stimulation conditions.

### Kinematic feature extraction and quantification (NERVTEX Co. Ltd.)

**Figure 1:**
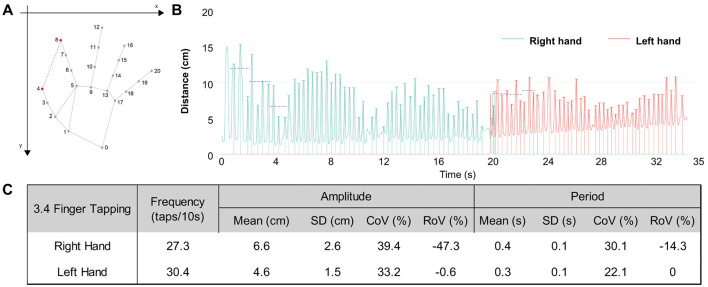
AI-based kinematic extraction and quantification of finger-tapping performance (MDS-UPDRS III, item 3.4). **(A)** Hand pose estimation using a 21-point skeletal model. Finger-tapping amplitude is defined as the frame-wise Euclidean distance between the thumb fingertip (point 4) and index fingertip (point 8), illustrated by the red dashed segment. **(B)** Time-series trajectories of tapping amplitude for the right and left hands. Each peak corresponds to a tapping cycle. The right hand shows larger amplitudes but greater fluctuation across time, whereas the left hand demonstrates smaller but more consistent movements. **(C)** Quantitative kinematic metrics extracted for each hand: frequency (taps/10 s), amplitude (mean, SD, CoV%, RoV%), and period (mean, SD, CoV%, RoV%). The right hand displays higher amplitude but poorer stability, reflected by larger CoV% and more negative RoV%, indicating declining performance over successive cycles. The left hand exhibits lower amplitude but better stability, with consistently smaller CoV% and near-zero RoV%.

Taking finger tapping (item 3.4) of one real patient as a representative example, each hand was tracked using 21 skeletal points. Movement quantification was based on the time-varying opening amplitude, defined as the distance between the thumb fingertip (point 4) and the index fingertip (point 8; Figure 1A). For each tapping cycle, both amplitude (maximum opening per cycle) and period (time required to complete one cycle) were extracted (Figure 1B).

In this analysis (Figure 1C), the right hand demonstrated a larger overall amplitude but exhibited poorer movement stability, as indicated by higher CoV (%) for both amplitude and period. Additionally, the later cycles showed notable reductions in amplitude and speed, with a negative RoV (%) of amplitude and period. In contrast, the left hand had smaller overall amplitude but showed greater stability, with lower CoV (%) for both amplitude and time.

### Levodopa produces broad motor gains with stabilization of upper-limb performance

**Figure 2:**
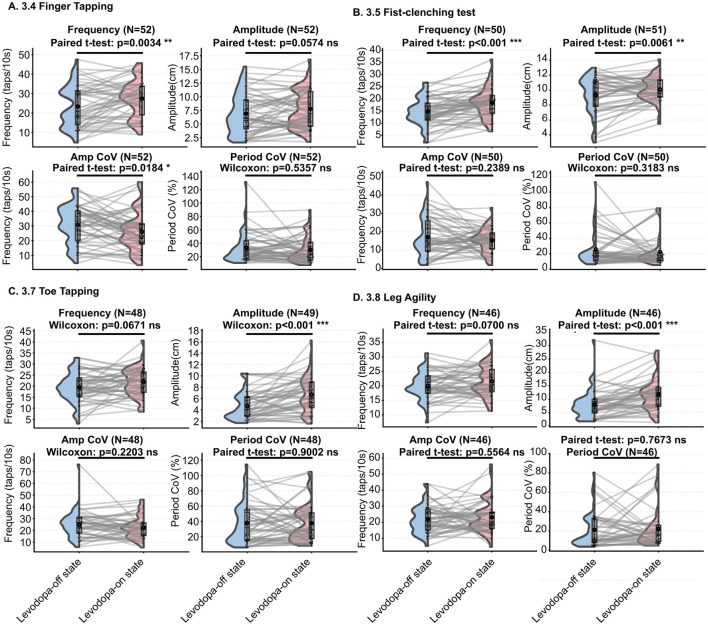
AI-based kinematic analysis of Levodopa effects on bradykinesia-related motor tasks (Levodopa-off vs. Levodopa-on). **(A)** Item 3.4 Finger Tapping. Levodopa increased tapping frequency significantly (*p* = 0.0034) and produced a trend toward higher amplitude (ns). Amplitude CoV/% decreased significantly (*p* = 0.0184), indicating improved movement stability. **(B)** Item 3.5 Fist-Clenching Test. Levodopa resulted in robust increases in frequency (*p* < 0.001) and amplitude (*p* = 0.0061). Variability metrics (Amplitude CoV/% and Period CoV/%) showed no significant change. **(C)** Item 3.7 Toe Tapping. Amplitude improved markedly (*p* < 0.001), while stability metrics (Amplitude CoV/% and Period CoV/%) remained statistically unchanged. **(D)** Item 3.8 Leg Agility. Levodopa produced significant increases in movement amplitude (*p* < 0.001), whereas frequency changes were not significant. Both Amplitude CoV/% and Period CoV/% showed minimal non-significant changes, highlighting limited effects on lower-limb movement stability. Significance thresholds: *p < 0.05; **p < 0.01; ***p < 0.001.

AI-based kinematic analysis demonstrated that levodopa administration led to clear improvements across both upper- and lower-limb motor tasks, reflected by increased speed and amplitude (Levodopa-on vs. Levodopa-off, Figure 2).

Regarding movement stability, both the Amplitude CoV (%) and Period CoV (%) of the upper limb decreased after medication, indicating that Levodopa improved the regularity of upper-limb movements (3.4 Finger Tapping Amplitude CoV (%) showed statistical significance).

In the lower limbs, decrease in Amplitude CoV (%) was observed for toe tapping, whereas stability metrics for leg agility showed minimal change, and none of the variability-related measures reached statistical significance. Detailed values are presented in Table 3.

**Table 3:** AI-derived kinematic features (mean ± SD) for MDS-UPDRS Part III items 3.4, 3.5 (upper limb) and 3.7, 3.8 (lower limb) comparing Levodopa-off and Levodopa-on states.

**Variable**	**Frequency (taps/10 s)**		**Amplitude (cm)**		**Amplitude CoV (%)**		Period CoV (%)	
	**Levodopa- off state**	**Levodopa- on state**	**Levodopa- off state**	**Levodopa- on state**	**Levodopa- off state**	**Levodopa- on state**	**Levodopa- off state**	**Levodopa- on state**
3.4 finger tapping	23.36 ± 10.83	27.17 ± 9.62	6.9 ± 3.72	7.72 ± 3.48	30.86 ± 12.86	25.74 ± 13.51	33.47 ± 22.66	30.14 ± 20.06
3.5 Fist-clenching test	14.77 ± 5.85	18.81 ± 6.44	9.31 ± 2.49	10.19 ± 1.69	17.15 ± 10.85	14.9 ± 6.63	26.09 ± 21.96	21.98 ± 9.12
3.7 toe tapping	19.44 ± 6.78	21.28 ± 5.45	4.88 ± 2.74	6.54 ± 3.26	24.66 ± 11.52	22.98 ± 9.56	38.75 ± 26.23	38.91 ± 27.55
3.8 leg agility	19.79 ± 5.29	21.09 ± 5.45	7.76 ± 5.49	11.65 ± 6.59	22.15 ± 8.8	22.91 ± 10.08	23.28 ± 23.3	23.27 ± 20.74

Values are presented as mean ± standard deviation (SD). Extracted kinematic parameters include movement frequency (taps/10 s), amplitude (cm), amplitude coefficient of variation (Amp CoV, %), and period coefficient of variation (Period CoV, %). Statistical analyses comparing Levodopa-off and Levodopa-on states are presented in Figure 2.

### DBS improves upper-limb motor output but yields limited stabilization in lower-limb tasks

**Figure 3:**
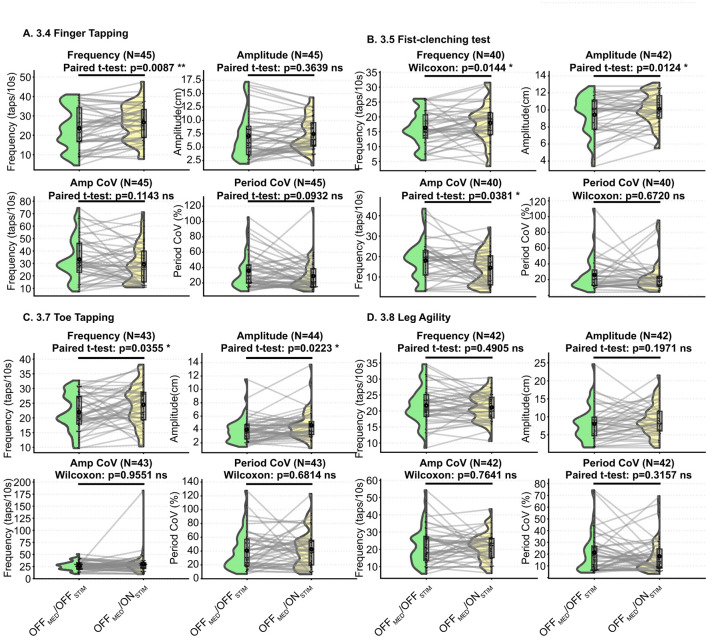
AI-based kinematic analysis of DBS effects on bradykinesia-related motor tasks (OFF_MED_/OFF_STIM_ vs. OFF_MED_/ON_STIM_). **(A)** Item 3.4 Finger Tapping. DBS significantly increased tapping frequency (*p* = 0.0087) but did not significantly change amplitude. Variability metrics (Amplitude CoV/% and Period CoV/%) showed non-significant downward trends. **(B)** Item 3.5 Fist-Clenching Test. DBS improved movement frequency (*p* = 0.0144) and amplitude (*p* = 0.0124). Amplitude CoV/% decreased significantly (*p* = 0.0381), whereas Period CoV/% showed no significant change. **(C)** Item 3.7 Toe Tapping. Lower-limb tapping frequency (*p* = 0.0355) and amplitude (*p* = 0.0223) both increased following DBS activation. However, stability indices (Amplitude CoV/% and Period CoV/%) remained unchanged (ns). **(D)** Item 3.8 Leg Agility. DBS did not produce significant changes in frequency or amplitude for leg agility. Variability metrics also showed no meaningful differences. Significance thresholds: *p < 0.05; **p < 0.01; ***p < 0.001.

Following DBS activation (OFF_MED_/OFF_STIM_ vs. OFF_MED_/ON_STIM_), upper-limb motor tasks demonstrated clear increases in both movement speed and amplitude. In the lower limbs, item 3.7 toe tapping also showed improved performance with greater frequency and amplitude, whereas changes in item 3.8 leg agility were minimal across all measured kinematic parameters (Figure 3).

Regarding movement stability, upper-limb variability (Amplitude Cov/% and Period Cov/%) exhibited an overall decreasing trend, indicating more regular performance (3.5 Fist-clenching test Amplitude CoV (%) showed statistical significance). In contrast, lower-limb stability did not improve after DBS activation, with mean variability metrics showing a trend toward higher values, although these changes did not reach statistical significance. Detailed values are presented in Table 4.

**Table 4:** AI-derived kinematic features (mean ± SD) for MDS-UPDRS Part III items 3.4, 3.5 (upper limb) and 3.7, 3.8 (lower limb) comparing OFF_MED_/OFF_STIM_ and OFF_MED_/ON_STIM_ states.

**Variable**	**Frequency (taps/10 s)**		**Amplitude (cm)**		**Amplitude CoV (%)**		**Period CoV (%)**	
	**OFF**_MED_ **/ OFF**_STIM_	**OFF**_MED_ **/ ON**_STIM_	**OFF**_MED_ **/ OFF**_STIM_	**OFF**_MED_ **/ ON**_STIM_	**OFF**_MED_ **/ OFF**_STIM_	**OFF**_MED_ **/ ON**_STIM_	**OFF**_MED_ **/ OFF**_STIM_	**OFF**_MED_ **/ ON**_STIM_
3.4 finger tapping	23.18 ± 11.0	26.7 ± 9.48	7.09 ± 4.18	7.38 ± 3.1	33.79 ± 17.34	29.03 ± 15.1	35.22 ± 23.13	28.55 ± 19.22
3.5 fist-clenching test	16.1 ± 5.71	17.92 ± 6.53	9.42 ± 2.31	10.14 ± 1.94	17.89 ± 9.86	15.8 ± 10.68	25.94 ± 20.3	26.95 ± 25.77
3.7 toe tapping	21.55 ± 6.63	23.78 ± 7.57	3.97 ± 1.95	4.63 ± 2.30	26.48 ± 9.84	30.22 ± 24.87	41.92 ± 27.26	43.57 ± 27.12
3.8 leg agility	21.63 ± 5.88	20.98 ± 4.53	8.08 ± 4.84	8.64 ± 4.51	22.25 ± 10.96	26.26 ± 28.67	21.2 ± 16.99	22.27 ± 21.84

Values are presented as mean ± standard deviation (SD). Extracted kinematic parameters include movement frequency (taps/10 s), amplitude (cm), amplitude coefficient of variation (Amp CoV, %), and period coefficient of variation (Period CoV, %). Statistical analyses comparing OFF_MED_/OFF_STIM_ and OFF_MED_/ON_STIM_ states are presented in Figure 3.

### Medication more effectively improves lower-limb amplitude

**Figure 4:**
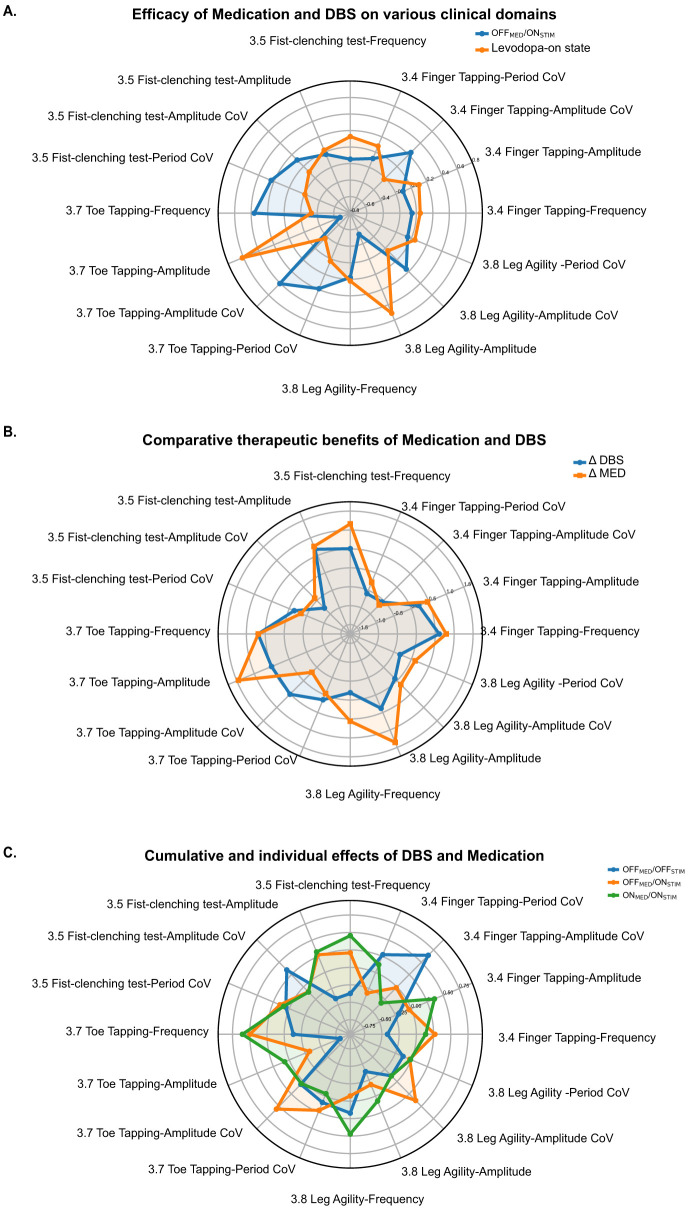
Radar-plot visualization of multidimensional kinematic responses to Levodopa and DBS across bradykinesia-related tasks. **(A)** Comparison between Levodopa-on and OFF_MED_/ON_STIM_ conditions. Radar plots constructed from each kinematic metric illustrate that Levodopa and DBS produce broadly comparable enhancements in upper-limb motor performance (items 3.4 finger tapping and 3.5 fist-clenching). In contrast, DBS is associated with higher frequency in item 3.7 toe tapping and lower amplitude in both lower-limb tasks (items 3.7 and 3.8). Regarding variability measures (CoV/%), the most notable differences appeared in item 3.7. **(B)** Change-score comparison of medication versus DBS effects. Radar plots derived from within-subject change scores (Levodopa-off → Levodopa-on vs. OFF_MED_/OFF_STIM_ → OFF_MED_/ON_STIM_) demonstrate a similar pattern: Levodopa produces more pronounced improvements in lower-limb amplitude, particularly for item 3.7 toe tapping and item 3.8 leg agility. **(C)** Combined and individual effects of DBS and medication across postoperative states. When the three stimulation/medication states (OFF_MED_/OFFSTIM, OFF_MED_/ON_STIM_, ON_MED_/ON_STIM_) are visualized together, the ON_MED_/ON_STIM_ condition shows the most favorable multidimensional motor profile. This combined treatment state is characterized by faster movement frequency, larger movement amplitude, and lower variability (CoV/%) across tasks despite some minor deviations in individual items.

The radar plots in Figure 4A (Levodopa-on vs. OFF_MED_/ON_STIM_), generated using the absolute values of each kinematic metric, showed that DBS and medication produced broadly comparable effects on upper-limb performance (items 3.4 and 3.5). In contrast, for lower-limb tasks, DBS was associated with higher frequency in item 3.7 but markedly reduced amplitude in both item 3.7 and item 3.8. Regarding variability measures (CoV/%), the most notable differences appeared in item 3.7, where DBS resulted in higher amplitude and period coefficients of variation, indicating less stable than those observed with medication.

Radar plots based on change scores (Levodopa-off to Levodopa-on vs. OFF_MED_/OFF_STIM_ to OFF_MED_/ON_STIM_) yielded similar conclusions, with medication showing a more pronounced improvement in lower-limb amplitude (Figure 4B). Although the differences in variability measures (CoV) were no longer as prominent, significant differences in lower-limb amplitude were still evident, with medication demonstrating a greater improvement compared to DBS.

Furthermore, when the three postoperative stimulation states were visualized using the same radar-plot framework (Figure 4C), the combination of medication and stimulation produced the most favorable overall performance profile, characterized by faster movement frequency, larger amplitude, and lower coefficients of variation for both frequency and amplitude, despite some minor deviations in individual items.

Further analysis (Table 5, Levodopa-on vs. OFF_MED_/ON_STIM_) showed that Levodopa produced a significantly greater increase in lower-limb movement amplitude, including item 3.7 toe tapping (6.54 ± 3.26 cm vs. 4.63 ± 2.30 cm, *p* < 0.001) and item 3.8 leg agility (11.65 ± 6.59 cm vs. 8.64 ± 4.51 cm, *p* < 0.05).

**Table 5:** AI-derived kinematic features (mean ± SD) for MDS-UPDRS Part III items 3.4, 3.5 (upper limb) and 3.7, 3.8 (lower limb) comparing Levodopa-on and OFF_MED_/ON_STIM_ conditions

**Variable**	**Frequency (taps/10 s)**		**Amplitude (cm)**		**Amplitude CoV (%)**		**Period CoV (%)**	
	**Levodopa-on state**	**OFF**_MED_ **/ON**_STIM_	**Levodopa-on state**	**OFF**_MED_ **/ ON**_STIM_	**Levodopa-on state**	**OFF**_MED_ **/ON**_STIM_	**Levodopa-on state**	**OFF**_MED_ **/ON**_STIM_
3.4 finger tapping	27.17 ± 9.62	26.7 ± 9.48	7.72 ± 3.48	7.38 ± 3.1	25.74 ± 13.51	29.03 ± 15.1	30.14 ± 20.06	28.55 ± 19.22
3.5 fist-clenching test	18.81 ± 6.44	17.92 ± 6.53	10.19 ± 1.69	10.14 ± 1.94	14.9 ± 6.63	15.8 ± 10.68	21.98 ± 19.12	26.95 ± 25.77
3.7 toe tapping	21.28 ± 6.72	23.78 ± 7.57	6.54 ± 3.26	4.63 ± 2.30	22.98 ± 9.56	30.22 ± 24.87	38.75 ± 26.23	43.57 ± 27.12
3.8 leg agility	21.09 ± 5.45	20.98 ± 4.53	11.65 ± 6.59	8.64 ± 4.51	22.91 ± 10.08	26.26 ± 28.67	23.27 ± 20.74	22.27 ± 21.84

Values are presented as mean ± standard deviation (SD). Extracted parameters include movement frequency (taps/10 s), amplitude (cm), amplitude coefficient of variation (Amp CoV, %), and period coefficient of variation (Period CoV, %). Statistical comparisons were conducted between Levodopa-on and OFF_MED_/ON_STIM_ states. Significant differences were observed in the amplitude of item 3.7 (toe tapping) and item 3.8 (leg agility) (*p* < 0.001 and *p* < 0.05, respectively). Only statistically significant *p*-values are reported; non-significant comparisons are not shown.

## Discussion

Conventional UPDRS-III scores can provide insights into improvements in PD symptoms from either medication or DBS. However, they are insufficient to capture the specific dimensional and detailed differences between the therapeutic effects of medication and deep brain stimulation (DBS). This limitation has been highlighted in previous reports, which suggest that ordinal clinical scoring lacks the sensitivity needed to detect subtle changes in key kinematic dimensions, such as speed, amplitude, and stability (13).

In this study, AI-based kinematic analysis allowed for a more granular examination of motor performance, revealing differences across specific motor dimensions in how levodopa and STN-DBS affect bradykinesia. Levodopa produced broad motor improvements across both upper and lower limbs, with significant increases in movement speed and amplitude. These findings align with the well-established dopaminergic restoration of cortico-striatal function, which enhances movement vigor and reduces bradykinesia (14). Importantly, levodopa demonstrated superior efficacy in improving lower-limb amplitude, particularly for toe tapping and leg agility, where amplitude gains were statistically significant. This preferential effect may reflect the strong dopaminergic dependence of axial and lower-limb bradykinesia compared with DBS (15), whose stimulation field may only partially modulate networks governing lower- limb control. On the other hand, DBS enhanced motor output in the upper limbs, where both speed and amplitude improved significantly after stimulation. These effects are consistent with prior findings indicating that STN-DBS effectively alleviates limb bradykinesia by modulating pathological oscillatory activity within the basal ganglia-thalamo-cortical circuit (16, 17). However, the amplitude improvements induced by DBS in the lower limbs were substantially smaller than those elicited by Levodopa. One possible explanation is the differential connectivity of the STN motor territory: while upper-limb motor representations are densely mapped within the dorsal STN, lower-limb representations are more diffuse and anatomically variable, which may reduce the uniformity of stimulation effects (18, 19).

Another key finding from this study is that both Levodopa and DBS improved the stability of upper-limb movements, reflected by a decrease in the coefficient of variation (CoV/%). However, the impact on lower-limb stability was more limited. This observation supports prior literature suggesting that lower-limb bradykinesia involves more complex integration across locomotor regions of the brainstem and spinal circuits, which may be less responsive to both dopaminergic therapy and STN stimulation (20, 21). This mechanistic difference may explain the minimal stabilization observed in leg agility, even when amplitude and speed improved. For DBS specifically, stability improvements were largely confined to the upper limbs. In the lower limbs, variability metrics, including the coefficient of variation (CoV%), trended toward higher values for item 3.7 (toe tapping) and item 3.8 (leg agility) when comparing the OFF_MED_/OFF_STIM_ vs. OFF_MED_/ON_STIM_ conditions (Table 4), although these differences were not statistically significant. These findings suggest that while STN-DBS effectively enhances movement vigor, its ability to regularize rhythmic lower-limb movements may be limited, at least during the early postoperative period. This pattern is consistent with observations reported in previous studies (22). Two mechanistic explanations may account for the relatively weaker lower-limb response to DBS. On one hand, the STN does not directly modulate mesencephalic locomotor regions (23), such as the pedunculopontine nucleus (PPN), which are crucial for rhythmic gait-related movements. This may limit DBS-induced improvements in cycle regularity and amplitude. In addition, all DBS recordings in this study were obtained 1 month after surgery—a time point when peri-elecrode edema had not fully resolved, and residual microlesion effects may have influenced DBS effects on motor outcomes (24). Consequently, the findings presented here reflect early-phase DBS effects, superimposed by stimulation and microlesion effects, rather than the outcome of fully established DBS stimulation. Furthermore, programming is often prioritized based on upper limb motor responses, potentially introducing a bias where lower limb motor improvements appear less pronounced than those in the upper limbs. Prior studies have shown that ventral STN stimulation and lower frequencies may preferentially improve lower-body parkinsonism (25), suggesting that the relatively weaker lower-limb response observed here may partly reflect programming and targeting strategies rather than fundamental mechanistic differences. Consistent with this view, frequency-based programming approaches have been reported to differentially influence lower-limb and gait-related features compared with other motor domains (26), further supporting the role of stimulation location and parameter selection in shaping symptom-specific DBS effects.

In summary, the findings of this study highlight distinct therapeutic strengths: levodopa excels in restoring amplitude and vigor, especially for lower-limb bradykinesia; DBS is effective in enhancing upper-limb performance, with modest effects on lower-limb amplitude and limited impact on movement stability. These observations may help refine individualized treatment strategies, particularly for patients whose primary disability lies in lower-limb bradykinesia or axial motor impairment.

This retrospective study relied on available follow-up videos, resulting in missing data and variable sample sizes for certain tasks. In addition, clinicians scoring the videos were not fully blinded to treatment condition, as contextual cues inherent to the recordings (e.g., visible stimulation effects or medication-related motor changes) could potentially be inferred, and observer bias therefore cannot be entirely excluded. Furthermore, all DBS assessments were conducted during the acute postoperative activation phase, which likely underestimates the long-term effects of stimulation. Finally, although the AI extraction system captured detailed kinematic features, further refinement may be needed to optimize sensitivity to subtle lower-limb motor fluctuations. These findings underscore the complementary mechanisms of medication and DBS and highlight the potential value of AI-based tools for precise, multidimensional motor assessment in Parkinson's disease.

## Data Availability

The raw data supporting the conclusions of this article are available from the corresponding author upon reasonable request.
